# Latin American Asylum Seekers in Spain: Acculturation Preferences and Related Psychosocial Variables

**DOI:** 10.5334/irsp.1113

**Published:** 2026-05-14

**Authors:** Marisol Navas, María Sánchez-Castelló, Anna Maria Meneghini, Pablo Pumares, Antonio J. Rojas

**Affiliations:** 1 Universidad de Almería, CEMyRI, Spain; 2 Universitàdi Verona, Ciesseci, Italy

**Keywords:** Latin American asylum seekers, acculturation process, intergroup attitudes, quality of contact, social support

## Abstract

The aim of this study was to explore the Latino American asylum seekers’ acculturation preferences in Spain and their relationship with psychosocial variables (stereotypes, emotions, quality of contact, perceived discrimination and social support), differentiating between public and private domains of the Relative Acculturation Extended Model (RAEM). Ninety-eight asylum seekers (44.9% females) answered a questionnaire. Results show that they prefer assimilation in public domains and integration in private domains, showed positive stereotypes and emotions towards Spaniards, perceived a pleasant contact, a low level of discrimination, and moderate-high social support. Different significant predictors emerged: emotions and family support (in public domains) and family support (in private domains) for maintenance preferences; and competence and quality of contact (in public domains) and friends support (in private domains) for adoption preferences.

In recent years, the number of people forced to flee their countries has increased steadily. According to the United Nations High Commissioner for Refugees (UNHCR, 2023), the number of people in refugee or refugee-like situations and other persons in need of international protection has increased from around 12 million in 2013 to almost 35 million in 2022. While it is usually less developed countries that are primarily affected, since the so-called *refugee crisis* of 2015, stemming from the Syrian war, the European Union (EU) (at 27 Member States, excluding the United Kingdom) has received 6.88 million first asylum applications. The challenge facing European asylum systems in integrating these people has become a hotly debated topic in the EU.

Although Spain has become one of the main incoming countries of important migratory flows since the mid-1990s, there was not a great tradition of receiving significant flows of refugees (Pumares et al., 2021). However, in recent years this has changed dramatically. Since 2019, Spain has become the third most popular country in the EU in terms of asylum applications, behind only Germany and France. According to Eurostat (2023), between 2019 and 2023, it received 551,990 applications for international protection, 162,420 of them in 2023.

Despite this increase in asylum applications in Europe and Spain, most psychosocial research on the factors involved in intergroup relations focuses on migrants in general, without distinguishing between immigrants and refugees, or else focuses on different groups of immigrants. Psychosocial research on acculturation has extensively recognized, almost since its inception, the existence of diverse migrant groups and varying motives for migration. It also highlights how this variability influences the different acculturation orientations that migrants develop, as well as the degree of adaptation they achieve within receiving societies. Berry (1997) identified three factors to describe the variety of cultural groups in plural societies: voluntariness, mobility, and permanence. Thus, some cultural groups engage in the acculturation process voluntarily (e.g., immigrants seeking better opportunities or international students), while others do so involuntarily (e.g., refugees, asylum seekers, or forcibly displaced persons). Regarding mobility, some groups relocate to a new place (migrants or refugees), while others, despite remaining in their own territory, must acculturate to a different or majority culture (e.g., indigenous peoples). Finally, permanence distinguishes between groups that settle relatively permanently and those who return, plan to return, or are forced to return to their societies of origin (e.g., seasonal workers, international students, or asylum seekers facing deportation). These factors can determine the psychological experience, the acculturation process, and the ultimate adaptation of these groups (Berry & Sam, 1997). Clearly, acculturation is not a uniform experience; rather, it is shaped by these variables. Specifically, for the purposes of this research, acculturation is shaped by whether the migration is forced (e.g., due to conflict or persecution) or voluntary (e.g., for work or education).

In contrast to immigrants, refugees, asylum seekers, and stateless people worldwide, are those people who had to leave their home country as a result of persecution, conflict, violence, human rights violations and events seriously disturbing public order (UNHCR, 2023). The reasons why refugees migrate (e.g., wars and conflicts) may therefore be profoundly different from the reasons why immigrants leave their country of origin and these reasons, for refugees, imply potentially more pronounced effects in terms of culture shock and the likelihood of experiencing mental health problems (Sheikh & Anderson, 2018).

This may be the reason why the few studies conducted with asylum seekers and refugees as participants have mainly turned to the analysis of widespread problems among them, such as mental health problems resulting from trauma (e.g., Hollifield et al., 2002; van der Boor et al., 2020). On the contrary, there is a shortage of studies that address the acculturation process of refugees and are concerned with how and to what extent the key factors of intergroup relations (i.e., stereotypes, emotions towards outgroup members, quality of contact, perceived discrimination, social support, etc.) influence these processes.

One of the contextual factors that influence the acculturation process is whether migration is voluntary or involuntary (Maehler & Daikeler, 2024). These authors point out that changes in cultural identity may depend on whether immigration was voluntary or involuntary, as legal, social, and economic access in the country of residence can vary accordingly. Among the results of their meta-analysis, they found that although both voluntary (non-refugees) and involuntary immigrants identified strongly with their culture of origin and moderately with the country of residence, voluntary immigrants showed a higher level of identification with their culture of origin compared to involuntary immigrants. Ertorer (2016) notes that the degree of voluntariness and the level of contact with the host society differ between immigrants and refugees: immigrants may hold more positive attitudes toward interaction and change with the host society, whereas refugees may become involved in the acculturation process involuntarily, which may lead to greater difficulties. Additionally, as Berry (2006) suggests, other factors—such as group size, status, and the rights and resources available—differentiate the various groups living in plural societies and influence how they undergo the acculturation process.

In addition, some scholars have highlighted that the attitudes of host communities towards immigrants and refugees are also different, and that not all refugees are the same or elicit the same reactions in host communities (Abdelaaty & Steele, 2022; Kotzur et al., 2019; Wyszynski et al., 2020). Recent psychosocial research in some European countries, using the Stereotype Content Model (SCM, Fiske et al., 2002) or its extension and the BIAS Map (e.g., Cuddy et al., 2008), shows that the labels used to describe groups (e.g., immigrant vs. refugee; different immigrants vs. refugee groups) influence the stereotype content held about them, the emotions experienced and the behavioral intentions people have towards them (Kotzur et al., 2019). These perceptions and attitudes of host societies about refugees therefore also influence intergroup relations and attitudes, as well as the acculturation processes that these ethnic minorities develop in their new communities.

The present study aims to shed light on the understudied minority perspective refugees, and in particular on a specific group of asylum seekers in Spain, which has increased in recent years, i.e., asylum seekers of Latin American origin. We aim to analyze their preferences for acculturation as well as the psychosocial variables related to this process (i.e., stereotypes and emotions towards host members, quality of contact, perceived discrimination and social support). To the best of our knowledge, no study with these features has been specifically conducted in Spain.

## Acculturation process of ethnic minorities in host societies

Acculturation refers to the process of cultural transformation that occurs when two or more culturally distinct groups come into contact, and to the changes that people experience (“psychological acculturation”) in identity, attitudes, values and behaviors during that process (Berry, 1997). The literature has shown that this process is reciprocal: no cultural group remains unchanged following intercultural contact, and acculturation is a two-way interaction (Sam & Berry, 2010). The acculturation process from a psychosocial perspective has been studied through two-dimensional models (e.g., Berry, 1997; Bourhis et al., 1997), so-called because they consider the two main factors encountered during acculturation: “cultural maintenance”—the extent to which minorities maintain their original cultural traditions or identity—, and “cultural adoption” (or “contact and participation”)—the extent to which they adopt cultural elements of the host society (or have contact and participate in the larger society along with other ethnocultural groups). The simultaneous consideration of these two factors or dimensions leads to four ways of solving or overcoming the acculturation process. In the case of minorities, these acculturation options are called integration (maintaining and adopting), assimilation (adopting and not maintaining), separation (maintaining and not adopting) and marginalization (neither maintaining nor adopting).

Based on the classical models, the Relative Acculturation Extended Model (RAEM; Navas et al., 2005; Navas & Rojas, 2010), a more recent model developed in the Spanish context, considers that the acculturation process of ethnic minorities can be analyzed at two different levels: the real plane (i.e., perceptions or strategies put into practice—the way in which minorities are acculturating), and the ideal plane (i.e., acculturation preferences—the way in which minorities would like to acculturate, if they could choose). In addition, the RAEM considers different domains of sociocultural reality, which can be grouped into public/peripheral (e.g., political, social welfare, work, economic) and private/central (e.g., social, family, religion and values) domains. Different acculturation options (e.g., integration, assimilation, separation or marginalization) can be implemented (or perceived) and preferred in these life domains.

This idea of decomposing acculturation into specific domains, in which people can choose different acculturation options, has been previously supported by numerous authors (e.g., Arends-Toth & Van de Vijver, 2004; Berry & Sam, 1997; Bourhis et al., 1997; Horenczyk, 1996). However, unlike previous acculturation models, the RAEM positions domains as its analytical core and develops domain-specific measurement instruments. This distinction by domains allows the different acculturation strategies and preferences of minority groups to be captured depending on the specific life domain: the results of previous empirical studies show that migrant ethnic minorities tend to prefer integration in public domains, while they prefer separation in private ones (e.g., Arends-Toth & Van de Vijver, 2003, 2004; Navas et al., 2005; Navas & Rojas, 2010). Although the RAEM analyzes the acculturation process of ethnic minorities both from their own perspective (that is, how they acculturate and how they would like to acculturate) and from the perspective of the host society (that is, how they perceive migrants to be acculturating and how they would prefer them to acculturate), this study will consider only the perspective of the minority group (asylum seekers) regarding their acculturation process in Spain.

Research has examined the acculturation process of refugees and asylum seekers from diverse backgrounds and in various contexts, using qualitative (e.g., Colic-Peisker & Walker, 2003; Kämmer & Albert, 2023; Phillimore, 2011; Şafak-Ayvazoglu & Kunuroglu, 2021; Şafak-Ayvazoglu et al., 2020), quantitative (e.g., Ertorer, 2016; Roblain et al., 2017) or mix-methods (e.g., Bayram & Eryilmaz, 2025) approaches. However, most studies overlook the distinction between public and private acculturation domains, with Şafak-Ayvazoglu & Kunuroglu (2021) being the sole exception to incorporate this differentiation. In their study, they found that Syrian refugee university students in Turkey had opted for integration in the public sphere and separation in the private sphere.

Roblain et al. (2017) analyzed the acculturation preferences of Syrian and Iraqi asylum seekers in Belgium. Their study found that both groups exhibited a strong desire to adopt the host culture while simultaneously expressing a high desire to maintain their culture of origin (preferences for integration into the host society). This preference for integration in the acculturation process of refugees is also found in other studies with refugees of different origins in different host countries. Kämmer & Albert (2023) found that the former refugees (from Iraq, Iran and Togo) they interviewed in Germany had incorporated elements of both cultures. On the other hand, Bayram & Eryilmaz (2025) found that while integration is the primary strategy among Syrian refugees in Turkey (57%), this preference is significantly more pronounced among immigrants, particularly those of Russian origin (70%). Alternatively, Colic-Peisker & Walker (2003) analyzed the resettlement patterns of Bosnian refugees in Australia. They found evidence of integration, marginalization, and separation, with a relative separation from the dominant society being the most widespread strategy. Phillimore (2011) also identifies a trend toward the segregation (and marginalization) of refugees in the United Kingdom, driven by immigration policies characterized by a lack of support, negative attitudes, and discrimination toward asylum seekers and refugees, as well as by the absence of refugee community organizations.

## Psychosocial variables related to the acculturation process

Research on the acculturation process of ethnic minorities in host societies—mainly immigrants—has revealed the existence of a large number of psychosocial variables (e.g., individual, interpersonal, group and contextual) that may influence how immigrants prefer to acculturate and how they finally do so in the receiving societies. These related variables with acculturation process include, among others, intergroup attitudes (e.g., stereotypes and emotions), the quality of intergroup contact, perceived discrimination, and perceived social support.

Studies with the RAEM in Spain with immigrant minorities of different origins (e.g., Maghrebi and sub-Saharan, Navas et al., 2007; Romanian and Ecuadorian, Navas & Rojas, 2010), have shown that immigrants of different origins prefer a greater adoption of the host culture (assimilation or integration options) when they have positive stereotypes and emotions towards the host society, when contact with the latter is positive, and when they feel well-treated (or not discriminated against). These studies have also shown the importance of considering the acculturation domain (public vs. private) because the same acculturation options are not preferred in each domain. Thus, immigrants show a greater preference for adopting the host culture in public (vs. private) domains, and conversely, a greater preference for maintaining their culture of origin in private (vs. public) domains.

Research focusing on refugees as target persons in other European countries has also shown the relevance of these variables in the process of acculturation and adaptation of refugees. Thus, Lutterbach and Beelmann (2021) analyzed how refugees’ stereotypes towards host society members relate to the acculturation-relevant attitudes in their first phase of acculturation. They found that when the members of the host community (Germans) are evaluated as high in sociability (a stereotypical dimension of the extended SCM model, Leach et al., 2007) by the refugees, their perception of commonalities with the Germans and their orientations to adopt German culture are greater. In addition, these researchers highlighted that greater perceived everyday individual discrimination (i.e., perceiving unfair treatment because they belong to a socially devalued group) fuels the motivation to maintain the culture of origin (as the refugees probably tend to “take refuge” in their ethnic ingroup), while greater discrimination perceived at an institutional level (i.e., unequal treatment by governmental and social institutions and services, e.g. the police) is associated with a lower orientation to maintain the culture of origin.

In this vein, it was highlighted that a lower perception of discrimination facilitates refugees’ acculturation (Esses et al., 2017). In a context where refugees’ culture (Syrian refugees) bears similarities with that of the host society (Lebanon), Tohme et al. (2024) found that refugees’ low perceived discrimination (and low cultural distance) predicted high host acculturation (i.e., orientation towards the host society), as well as better psychological and socio-cultural adaptation. Similar results have been found by Safdar et al. (2024) studying the acculturation and adaptation process of Venezuelan refugees in Colombia: low perceived discrimination was associated with higher host orientation and psychological adaptation.

Moreover, the knowledge of the language of the host country (i.e., language competence) positively correlates to intergroup contact (Tip et al., 2019), facilitates integration (Esses et al., 2017), and crucially influences the success of cultural adaptation (Chen et al., 2008).

In addition to low discrimination and increased language proficiency, another element that can become a protective and facilitating factor in the acculturation process of refugees is the social support they perceive from different sources (e.g., family, friends, institutions, NGOs, etc.). Social networks are crucial for asylum seekers and refugees and protect them from mental disorders, which in turn leads to better adaptation in the host society (van der Boor et al., 2020). Kovács et al. (2023) found that perceived social support (along with post-traumatic stress and host country), among Ukrainian war refugees in Poland and Hungary, was a significant predictor of refugees’ integrative acculturation attitudes (i.e., attitudes towards simultaneous cultural maintenance and adoption in different spheres of life) in both countries. Although the two samples did not differ in their levels of post-traumatic stress, refugees in Poland perceived significantly more social support and displayed stronger integration attitudes than refugees in Hungary. Kovács et al. (2023) pointed to the greater number of Ukrainian people in Poland, even before the war, and the greater cultural and linguistic similarity of Ukraine and Poland (vs. Hungary) as potentially explanatory factors for this result.

## The present study

The present study was carried out in the province of Almería (southeast of Spain), which has a high percentage of immigrants (23.76% of the population is foreign-born according to the National Institute of Statistics, Instituto Nacional de Estadística [INE], 2024). The province specializes in intensive greenhouse agriculture, which is one of the main sources of employment for the immigrant population. Asylum seekers in the province thus find themselves in an environment where they can easily meet other immigrants, although not always compatriots, and where there are significant employment opportunities, although this is not always suited to their skills.

In this study we focus on Latin American asylum seekers. One of the main factors that explains the consolidation of Spain as one of the main European countries receiving refugees was the growing influx of Latin American asylum seekers. Initially, they were closely linked to the Venezuelan exodus triggered by the government of Nicolás Maduro, but they were soon followed by Colombia, Peru and several Central American countries, favored by the greater ease of access to Spanish territory and the anti-immigration policy of the US (Gabrielli et al., 2022). Thus, the composition of the asylum seekers in Spain according to origin differs greatly from that of the rest of the EU countries. It is characterized by the enormous prominence of those from Latin America (Spain has the vast majority) and by the comparatively low presence of applicants from Asia and the Middle East, which predominate in many other countries. This is an advantage in that they do not have to go through the laborious process of learning a new language, which often hampers the chances of labor market integration and social integration of applicants from elsewhere.

Spain has a specific reception program called the System for the Reception and Integration of Persons Seeking and Benefiting from International Protection (SAISAR), managed by the Ministry of Inclusion, Social Security and Migration. Those seeking international protection must express their willingness to apply for asylum in Spain during the first month of their stay and formalize it through an interview. The Office of Asylum and Refuge (OAR), which is part of the Ministry of the Interior, will then decide whether they are admissible within a month. If that is the case they can enter the first phase of the reception program—the initial assessment and referral phase—in which their situation and specific needs are analyzed. Those who are deemed to lack sufficient means to live, and only these, will be referred to a reception facility, where they will receive support of various kinds (e.g., language, psychological care, job orientation, etc.). This is the second phase (reception), lasting six months, during which they are not yet allowed to work. Finally, after six months or when the OAR gives a favorable decision on the asylum application, they enter the third phase (autonomy), in which they leave the facility and must achieve economic independence within another six months. This study focuses on asylum seekers who are in the reception phase (second phase).

The aim of this study is to analyze the acculturation preferences—both for the maintenance of the culture of origin and for the adoption of the host culture and differentiated by public and private domains contemplated by the RAEM—of Latin American asylum seekers in Spain. In addition, we explore the relationship between mentioned psychosocial variables (stereotypes, emotions, quality of contact, perceived discrimination, and social support) with variables of acculturation preferences. Hence, this paper contributes to casting light on the acculturation processes of refugees or asylum seekers in their new communities.

In our study, as previously mentioned, we differentiate between public (i.e., political, social welfare, work and economic) and private (i.e., social, family, religious and values) acculturation domains. To the best of our knowledge, there are no prior studies with refugee or asylum-seeking populations that analyze acculturation orientations using this distinction, with the exception of the study by Şafak-Ayvazoglu & Kunuroglu (2021). In this study they found that Syrian refugee university students in Turkey opted for integration in the public domains and for separation in the private domains. Based on this result and the results of the RAEM with migrants of different origins (Navas et al., 2007; Navas & Rojas, 2010; Rojas et al., 2014), we expected asylum seekers to prefer assimilation (i.e., adopt Spanish culture) or integration (i.e., maintain their culture of origin and adopt Spanish culture) acculturation options in the public RAEM domains (*Hypothesis 1*), while they were expected to prefer maintaining their culture of origin (and adopt less Spanish culture) more in private or central domains (i.e., separation or integration-separation options; *Hypothesis 2*). Previous studies have shown that some of the psychosocial variables included in the present study are related to the acculturation preferences of refugees/asylum seekers (e.g., Kovács et al., 2023; Lutterbach and Belmann, 2021; Safdar et al., 2024; Tohme et al., 2024), as well immigrants (Navas et al., 2007; Navas & Rojas, 2010). However, as there are no previous studies that analyze the effect of all the psychosocial variables included in this study on acculturation preferences differentiated by public and private domains of the RAEM, we approach these relationships in an exploratory way.

## Materials and Methods

### Participants

The study involved a group of 98 asylum seekers (44.9% females), aged between 19 and 68 years old (*M_age_* = 36.20; *SD* = 11.15) and residents in Almería (south of Spain), city and province (7.1% and 92.9% respectively). To determine the required sample size, a power analysis was conducted using G*Power software. An F-test was selected for a multiple linear regression model (*R²* deviation from zero) with seven predictors. To achieve a medium effect size (*f^2^* = 0.17) at a significance level of *p* = .05 and a power of .80, the analysis indicated that a minimum sample size of *N* = 92 participants was required to detect the proposed effect. All the participants were in their Temporary Reception phase in the SAISAR program, with an average stay in Spain of 10.31 months (*SD* = 3.87). According to the declared nationalities, they came from eight Latin American countries: Colombia (30.6%), Venezuela (30.6%), El Salvador (10.2%), Nicaragua (9.2%), Peru (9.2%), Honduras (7.1%), Cuba (2.0%), and Paraguay (1.0%), coinciding with the dominant provenances in Spain. Most participants had a secondary school qualification (63.3%), 30.6% a university degree and the remaining 6.1% a primary school diploma. In relation to marital status, 60.2% were married, 30.6% were single, 5.1% were divorced, and 2% were widowed. Not all of them declared who they were living with at the time of the interview (26.5%). Of the respondents, 37.8% were living with a partner and children, 16.3% with a partner, 7% with children only and 12.2% alone. Regarding participants’ religious beliefs, the majority declared to be Catholic (65.3%). Of the remaining, only 1% declared to be Protestant; the others did not answer this question (24.5%) or reported being indifferent to religion or agnostic (9.2%). Regarding religious practices, the mean score was 5.64 (*SD* = 3.24) on a scale ranging from 1 (*Not at all practising*) to 10 (*Very practising*). Finally, 75.6% of the asylum seekers had left their country of origin intending to move to Spain, and, although upon arrival 37.8% were uncertain about this, 48.0% intended to stay permanently in this country when they were interviewed. Owing to their origin, they were all fluent Spanish speakers.

### Variables and Instruments

The scales and items used are presented below. The estimated reliability coefficients of all the scales and subscales are included in Table 1.

**Table 1:** Estimated reliability coefficients and descriptive statistics.


	CRONBACH’S ALPHA	SPLIT-HALF (S-B)	*M*	*SD*

**Acculturation preferences**				

Maintenance preferences (public domains)	.81	.85	2.35	1.01

Maintenance preferences (private domains)	.63	.68	3.67	0.83

Adoption preferences (public domains)	.69	.74	3.97	0.69

Adoption preferences (private domains)	.75	.72	3.60	0.78

**Related psychosocial variables**				

Morality	.79	.80	4.06	0.54

Sociability	.81	.83	3.96	0.77

Competence	.77	.80	4.00	0.67

Emotions	.71	.83	4.10	0.55

Quality of contact	.61	.69	3.58	0.69

Perceived discrimination	.78	.82	1.97	0.66

Family support	.90	.94	5.67	1.55

Friends support	.93	.91	4.52	1.80


*Note*. S-B is Spearman-Brown procedure.

#### Acculturation Preferences

Following the RAEM (Navas et al., 2005; Navas & Rojas, 2010) 16 items were used to assess participants’ acculturation preferences. Preferences were measured in eight domains of life divided into public (i.e., political, social welfare, work, and economic), and private domains (i.e., social, family, religious, and values). For each domain, the participants were asked to indicate how much they would like to maintain their own culture and how much to adopt that of the host country (Spain). All the items had a five-point scale (1 = *Not at all* to 5 = *Very much*). The higher the score on the maintenance scale, the greater the preference for asylum seekers to maintain their customs of origin. The higher the score on the adoption scale, the greater the preference for asylum seekers to adopt Spanish customs.

#### Stereotypes

This variable was measured by means of a scale of 17 items selected from the works of Sayans-Jiménez et al. (2017) and López-Rodríguez et al. (2013). The participants indicated to what extent they believe that some characteristics describe the Spaniards on a five-point scale (1 = *Not at all* to 5 = *Very much*): honest, trustworthy, sincere, respectful (morality dimension); aggressive, malicious, harmful, treacherous, untruthful (immorality dimension); friendly, warm, likeable, kind (sociability dimension); and intelligent, able, competent, effective (competence dimension). Immorality items were inverted and averaged with morality items for morality dimension. The average was calculated for sociability and competence dimensions. The higher the score, the more intense the positive stereotyping of Spaniards.

#### Emotions

This variable was measured by the emotion subscale of the Prejudiced Attitude Test by Rojas-Tejada et al. (2012). Participants were presented with three items referring to positive emotions (i.e., admiration, sympathy, respect) and four items to negative emotions (i.e., mistrust, discomfort, insecurity, indifference) which they evaluated using a five-point scale (1 = *Not at all* to 5 = *Very much*). The items related to negative emotions were inverted in such a way that the higher the score, the higher the presence of positive emotions towards Spaniards.

#### Quality of contact

A revised version of the Islam and Hewstone (1993) scale by Cervantes et al. (2017) was used: ‘How would you rate the contact you have had or have with Spaniards?’ The response options, in the form of bipolar adjectives, referred to the following aspects: *involuntary-voluntary, superficial-close* (friendly), *equal-unequal, pleasant-unpleasant, competitive-cooperative*. After reversing two items, the higher the score, the more favorable the contact with Spaniards is perceived to be.

#### Social support

This variable was measured with eight items of the adapted version of the Multidimensional Scale of Perceived Social Support (Zimet et al., 1988). Two sources of perceived support have been assessed: from family (four items; e.g., ‘I get the help and emotional support I need from my family’) and from friends (four items; e.g., ‘My friends really try to help me’). Response options range from 1 (*Strongly disagree*) to 5 (*Strongly agree*). High scores on the total scale or on each subscale are indicative of greater perceived social support.

#### Perceived group discrimination

The measure used by Navas and Rojas (2010), which aims to capture the perception that people have of the existence of discrimination against immigrants, was used. When asked: ‘To what extent do you think that people from your country are treated worse here than Spaniards in the following aspects?’ the participants had to respond to seven different situations/areas: politics, social welfare, housing, work, social, media and religion. The response scale was a five-point scale (from 1 = *Not at all* to 5 = *Very much*).

#### Sociodemographic variables

Sex, age, educational level, country of birth, nationality and main activity before leaving the country of origin were measured.

### Procedure

Participants were contacted through the non-governmental organizations that manage the program SAISAR in the province of Almería (Red Cross and CEPAIM Foundation). All the Latin American participants who were in their Temporary Reception phase of were selected. The questionnaire, which included additional variables to those reported in this paper, was individually delivered to each participant by the researchers. Participants were informed by the researchers of the objectives of the study, the anonymous and confidential treatment of the data to be obtained, the voluntary nature of participation, their potential withdrawal from the study at any time, and the fact that their explicit consent to participate in the study was required. Before the participants gave their authorization to answer the questionnaire, the researchers emphasized the strictly academic and research nature of the study, as well as the lack of relationship between participating in answering the questionnaire and the resolution of their asylum application. The interviews were conducted by a young, white female postdoctoral researcher with no personal migration background. At the time, she identified herself strictly as a researcher from the University of Almería, emphasizing her lack of involvement in the legal or administrative management of the SAISAR program. The estimated average time to answer the questionnaire was 30 minutes. Participants received no monetary rewards for their participation. The study was approved by the Bioethics Committee of the authors’ university (UALBIO2025/028). All data necessary to reproduce the reported results are publicly available: https://osf.io/d2wq7/overview?view_only=e0beb2f7cb3a439baca8d65f855772ec.

## Results

Descriptive statistics and bivariate correlations for all the variables are given in Tables 1 and 2, respectively. Latin American asylum seekers score much lower on preferences to maintain their culture in public than in private domains (*t*(97) = –13.13, *p* < .001, Cohen’s *d* = 0.99) and slightly higher on preferences to adopt the host country’s culture in public than in private domains (*t*(97) = 5.32, *p* < .001, Cohen’s *d* = 0.69). In addition, maintenance scores are lower than adoption scores in public domains (*t*(97) = –12.72, *p* < .001, Cohen’s *d* = 1.26). However, there are no differences in maintenance and adoption scores in private domains (*t*(97) = 0.61, *p* = .271). These results indicate a preference for assimilation in public domains and for integration in private domains.

**Table 2:** Bivariate correlations.


	1	2	3	4	5	6	7	8	9	10	11

**1.** MP (public domains)	–										

**2.** MP (private domains)	.44**	–									

**3.** AP (public domains)	–.06	.09	–								

**4.** AP (private domains)	.13	.05	.57**	–							

**5.** Morality	–.02	.15	.32**	.32**	–						

**6.** Sociability	–.04	.00	.34**	.34**	.46**	–					

**7.** Competence	–.10	.08	.40**	.32**	.57**	.58**	–				

**8.** Emotions	–.27**	.06	.26**	.25*	.67**	.54**	.48**	–			

**9.** Quality of contact	–.01	.22*	.38**	.27**	.35**	.39**	.33**	.49**	–		

**10.** Perceived discrimination	.05	–.12	–.10	–.14	–.43**	–.31**	–.31**	–.44**	–.36**	–	

**11.** Family support	.25*	.41**	.21*	.21*	.21*	.03	.03	.08	.25*	–.07	–

**12.** Friends support	.08	.11	.27**	.36**	.30**	.25*	.10	.25*	.19	–.11	.34**


*Note*. MP: maintenance preferences; AP: adoption preferences. *p < .05; **p < .01.

Regarding other related psychosocial variables, Spaniards are perceived as moral, sociable and competent by asylum seekers (high scores on these variables) and elicit positive emotions (high score). These results, taken as a whole, show a positive general attitude of the participants towards Spaniards. In addition, the quality of the contact is good (moderate-high score) and they perceive a very low level of discrimination by Spaniards (moderate-low score). Asylum seekers perceive support from their families and friends (moderate-high scores).

To explore the possible relationship of the variables included in the study with acculturation preferences, four multiple linear regression analyses were performed using maintenance and adoption preferences (in public and private domains) as outcome variables and stereotypes, emotions, quality of contact, and family and friends support as predictor variables. The results are presented in Table 3. In Model 1, using maintenance preferences in public domains as outcome variable (*F*(7, 90) = 3.12, *p* < .01, *R²*_adjusted_ = .13), emotions (*β* = –.52) and family support (*β* = .21) were statistically significant predictors. In Model 2, using maintenance preferences in private domains as outcome variable (*F*(7, 90) = 3.12, *p* < .01, *R²*_adjusted_ = .13), only family support (*β* = .37) was a statistically significant predictor. In Model 3, using adoption preferences in public domains as outcome variable (*F*(7, 90) = 4.98, *p* < .001, *R²*_adjusted_ = .22), competence (*β* = .28) and quality of contact (*β* = .25) were statistically significant predictors. In Model 4, using adoption preferences in private domains as outcome variable (*F*(7, 90) = 4.47, *p* < .001, *R²*_adjusted_ = .20), only friends support (*β* = .25) was a statistically significant predictor. Despite the relatively low *R²*_adjusted_ values, it is important to note the theoretical consistency of the predictors that were found to be statistically significant, as these differ across acculturation preferences and domains.

**Table 3:** Multiple regression analysis with maintenance and adoption preferences as dependent variables.


	MAINTENANCE PREFERENCES				ADOPTION PREFERENCES			
	
	MODEL 1 PUBLIC DOMAINS		MODEL 2 PRIVATE DOMAINS		MODEL 3 PUBLIC DOMAINS		MODEL 4 PRIVATE DOMAINS	
			
	B	*β*	B	*β*	B	*β*	B	*β*

Constant	3.08		1.94		1.54		1.09	

Morality	0.45	.24	0.14	.09	0.05	.04	0.13	.09

Sociability	0.20	.15	–0.11	–.10	0.07	.08	0.21	.21

Competence	–0.16	–.11	0.07	.06	0.29	.28*	0.15	.13

Emotions	–0.97	–.52***	–0.11	–.07	–0.15	–.12	–0.15	–.11

Quality of contact	0.12	.08	0.19	.16	0.25	.25*	0.11	.10

Family support	0.14	.21*	0.20	.37***	0.03	.07	0.04	.07

Friends support	0.01	.02	–0.02	–.04	0.06	.17	0.11	.25*

**Adjusted*R^2^***	.13		.13		.22		.20	


*Note.* *p < .05; **p < .01; ***p < .01.

All the analyses in this study were carried out with SPSS Statistics 29.0.

## Discussion

The first aim of this study was to explore Latino American asylum seekers’ acculturation preferences for the maintenance of the culture of origin and for the adoption of the host culture in the new host community, differentiating between public and private domains. In general, Latin American asylum seekers prefer assimilation (to adopt Spanish customs and not to maintain those of their countries of origin) in public domains of the RAEM (e.g., political, social welfare, work, and economic domains), as we had expected (*Hypothesis 1*). However, in private domains (e.g., social, family, religious, and values domains), integration is the preferred option of Latin American refugees.

Although we contemplated integration as a possible outcome in private domains, it is true that a greater preference for maintenance might be expected, as in the study with Romanian and Ecuadorian immigrants by Navas and Rojas (2010) and the study of Şafak-Ayvazoglu & Kunuroglu (2021) with Syrian refugee university students. One possible explanation for the reasons why there is not a clearer trend towards the culture of origin in private domains could be found in the specific characteristics of the minority group studied (i.e., asylum seekers). Asylum seekers are forced to leave a country, in many cases for political reasons, but always because it is not safe for them to remain. They are thus more inclined than others (e.g., migrants) to adopt the values and ways of understanding life and relationships of the host society (values such as equality, respect, peace, etc.). This trend toward integration manifests itself in most of the previously cited studies, which omit the distinction between the public and private domains (e.g., Bayram & Eryilmaz, 2025; Kämmer & Albert, 2023; Roblain et al., 2017).

Both the tendency to assimilate in public domains and the tendency to integrate in private domains could also be related to the linguistic and cultural similarities (or diminished cultural distance) of Latin American asylum seekers with the host society, even thanks to a high presence of compatriots who can provide social support. Kovács et al. (2023) found more integrative attitudes among Ukrainian refugees in Poland (vs. Hungary) and suggest these types of structural and environmental variables as explanatory. These factors could influence the acculturation process of asylum seekers.

On the other hand, this study explored the relationship between some psychosocial variables (e.g., stereotypes, emotions, quality of contact, perceived discrimination and social support) with preferences for cultural maintenance and adoption (differentiating between public and private domains of the RAEM). Our results show that some of these are related to acculturation preferences and that these relationships vary between public and private domains.

Family support perceived by asylum seekers seems to play a key role in maintenance preferences in both the public and the private domains. Perceived family support is related to the preference for maintaining the customs of origin. This positive relationship between the perception of family support and preferences for maintaining the culture of origin is in line with the results found by Blanc et al. (2022) in a sample of adolescents of Moroccan origin in Spain. Specifically, that study found that the perception of family support was related to a greater preference for maintenance through a higher quality of communication with parents (and greater family ethnic socialization on the part of mothers). In addition, in our study, participants’ positive emotions towards Spanish people are negatively related to preferences for maintenance in public domains. That is, asylum seekers’ positive emotions towards Spaniards are related to a lower preference to maintaining their customs regarding the political, social welfare, work, and economic domains.

Our results also show that the quality of contact with Spaniards is related to a greater preference of asylum seekers to adopt Spanish cultural elements in public domains. This relationship between positive contact and a greater preference for adopting (or identification with) the host country’s culture is in line with the results of previous studies (Navas et al., 2007; Navas & Rojas, 2010). It has also been observed that perceiving Spaniards as competent (a stereotypical dimension) is related to a greater preference for adoption in these domains (public). This result is partially in line with that obtained by Cuadrado et al. (2017) on Ecuadorian immigrants. The stereotypical perception that Ecuadorians had of Spaniards (in the dimensions of morality and competence) predicted their preference for adopting Spanish customs (through positive emotions), although this work did not differentiate between public and private acculturation domains. Asylum seekers of Latin American origin perceive Spaniards as competent (i.e., intelligent, able, competent, effective), a stereotypical dimension that derives from the perceived social status of the outgroup (Fiske et al., 2002), and this perception probably leads to a positive valuation of cultural elements of Spanish society and, therefore, to a greater preference to adopt those elements in public domains (i.e., political, social welfare, work, and economic domains).

Surprisingly, none of the other stereotypical dimensions (i.e., morality and sociability) towards the majority group were related to cultural maintenance or adoption, as occurred in previous studies (Lutterbach & Belmann, 2021). Nor does perceived discrimination appear to be a predictor of acculturation preferences in our study, as was the case in previous studies (Lutterbach & Belmann, 2021; Navas et al., 2007; Navas & Rojas, 2010; Safdar et al., 2024; Tohme et al., 2024). It is important to note that the levels of this variable in our study are very low, that is, Latin American asylum seekers do not feel they are treated any worse than Spaniards. One possible explanation for this result could lie in the more positive attitudes that Spaniards express towards immigrants from Latin American countries (e.g., Ecuadorians) compared with other groups (e.g., Romanians; Navas & Rojas, 2010), probably due to greater linguistic and cultural proximity, as well as the long-standing and close-knit historical and commercial ties. As noted, Latin Americans are one of the immigrant groups with the largest presence in Spain, and make up the largest group of asylum seekers (Gabrielli et al., 2022; Pumares et al., 2021).

Finally, perceived social support from friends is the only psychosocial variable that predicts asylum seekers’ preference for adopting Spanish cultural elements in private domains (i.e., social relationships, family, religion, and values). Although we do not have data on the origin of our participants’ friends, if we could assume that their circle of friends is also made up of Spaniards, this element could explain the greater preference to adopt the host culture in private domains. Previous research (e.g., Liamputtong & Kurban, 2018), has considered social support (from different backgrounds: friends, family, teachers, religion, etc.) as a protective factor in the acculturation and adaptation process of refugees. As Kovács et al. (2023, p. 3) summarize from other studies, ‘social support can mitigate refugees’ isolation and loneliness by enhancing the sense of belonging and facilitating integration into a new society’.

The results of our study have to be taken with caution due to some limitations. First of all, the correlational and cross-sectional design of the study does not allow causal relationships to be established between the variables studied. In addition, only asylum seekers from Latin America participated in this research. Future studies could analyze and compare the acculturation process of asylum seekers from other countries, which will present fewer similarities in language and cultural aspects with the host society, and towards whom there are evidently fewer positive stereotypes, emotions and behavioral tendencies. Finally, we cannot rule out that the interviewer’s Spanish background may have influenced participants’ responses (e.g., positive attitudes, low perception of discrimination). However, as mentioned in the methodology section, we attempted to minimize this potential bias by presenting the interviewer as an independent researcher unaffiliated with SAISAR’s legal/administrative processes, and lacking power to influence their situation.

Despite these limitations, we consider this research to be an important contribution to the study of intergroup relations and the process of acculturation from the perspective of asylum seekers in Spain, and of Latin American asylum seekers, in particular. Most studies have been conducted concerning migrants, whereas asylum seekers are people who have left their country of origin for reasons that differ from those of immigrants. Moreover, members of host communities show different attitudes towards them (Abdelaaty & Steele, 2022; Kotzur et al., 2019; Wyszynski et al., 2020). In Spain, the study by Sánchez-Castelló et al. (2022) showed that the attitudes of Spaniards towards refugees (without specifying their origin) are moderately positive (in terms of stereotypes, emotions and behavioral tendencies). In general, these attitudes are more positive than towards other minority groups (e.g., immigrants of African origin), found in previous studies. This implies the need to study the phenomenon of refugee acculturation processes separately from the immigrant population. In this vein, interventions to promote integration in the host community should take into account these differences and not be the same for migrants and asylum seekers.

Finally, a strength of this study is that it investigated acculturation preferences on the side of a group of asylum seekers who shared the language of the country of arrival and whose culture can be considered as being rather similar to that of the host community. Likewise, this study adds knowledge to acculturation processes by distinguishing between different life domains in which various acculturation options may be preferred. Finally, the paper sheds light on different psychosocial predictors of preferences for maintaining the culture of origin and adopting the host culture that are infrequent in asylum seeker studies.

## Positionality statement

This study was conducted by an interdisciplinary team of five White European faculty members (four Spanish and one Italian researcher; three female, two male) from the Interdisciplinary Collective of Social Studies and the Center for the Study of Migration and Intergroup Relations (Spain), and Ciesseci-Research Center for Community Development (Italy). Our expertise spans social psychology, human geography, international migrations and behavioral sciences methodologies.

The authors come from middle-class backgrounds and lack personal experience with international migration. Our collective career, however, has focused on intergroup relations and attitudes, acculturation processes, and migrant adaptation. We acknowledge our position of structural privilege and seek to counteract it through ongoing accountability to the communities we study, maintaining a continuous feedback loop with local migrant-led and pro-migrant associations, who participate as active collaborators in our projects.

All authors contributed to the theoretical framework and research design. Data collection via interviews was carried out by a young Spanish female postdoctoral researcher hired for the project, whose fieldwork was supervised by the authors to ensure consistency with the study’s methodological and ethical standards. Methodological specialists led the data analysis, while the entire team collaborated on the interpretation of results and their implications for social inclusion.
